# Socioeconomic Inequalities Persist Despite Declining Stunting Prevalence in Low- and Middle-Income Countries

**DOI:** 10.1093/jn/nxx050

**Published:** 2018-02-27

**Authors:** Inácio Crochemore M da Silva, Giovanny V França, Aluisio JD Barros, Agbessi Amouzou, Julia Krasevec, Cesar G Victora

**Affiliations:** 1 International Center for Equity in Health, Postgraduate Program in Epidemiology, Federal University of Pelotas, Brazil; 2 Johns Hopkins Bloomberg School of Public Health, Baltimore, MD; 3 Data and Analytics Section, Division of Data, Research and Policy, UNICEF, NY

**Keywords:** prevalence, rural population, socioeconomic factors, family characteristics, income, growth disorders, surveys and questionnaires

## Abstract

**Background:**

Global stunting prevalence has been nearly halved between 1990 and 2016, but it remains unclear whether this decline has benefited poor and rural populations within low- and middle-income countries (LMICs).

**Objective:**

We assessed time trends in stunting among children <5 y of age (under-5) according to household wealth and place of residence in 67 LMICs.

**Methods:**

Stunting prevalence was analyzed in 217 nationally representative Demographic and Health Surveys and Multiple Indicator Cluster Surveys from 67 countries with ≥2 surveys between 1993 and 2014. National estimates were stratified by wealth and area of residence, comparing the poorest 40% with the wealthiest 60%, and those residing in urban and rural areas. Time trends were calculated for LMICs by using multilevel regression models weighted by under-5 population, with stratification by wealth and by residence. Trends in absolute (slope index of inequality; SII) and relative (concentration index; CIX) inequalities were calculated.

**Results:**

Mean prevalences in 1993 were 53.7% in low-income and 48.2% in middle-income countries, with annual average linear declines of 0.76 and 0.72 percentage points (pp), respectively. Although similar slopes of declines were observed for the poorest 40% and wealthiest 60% groups in all countries (0.78 and 0.74 pp, respectively), absolute and relative inequalities increased over time in low-income countries (SII increased from –19.3% in 1993 to –23.7% in 2014 and CIX increased from *–*6.2% to –10.8% in the same period). In middle-income countries, socioeconomic inequalities remained stable. Overall, stunting prevalence decreased more rapidly among rural than for urban children (0.78 and 0.55 pp, respectively).

**Conclusions:**

The prevalence of stunting is decreasing. Poor-rich gaps are stable in middle-income countries and slightly increasing in low-income countries. Rural-urban inequalities are decreasing over time.

## Introduction

The short- and long-term consequences of child undernutrition are well known (1, 2). Stunting (low length or height for age) is a key indicator for assessing the nutritional status of children under the age of 5 y (under-5), being 1 of the 6 nutritional global targets established by World Health Assembly in 2012 (3) and a key indicator for the 2030 Sustainable Development Goals (SDGs) (4).

Stunting was estimated to affect 155 million children globally in 2016, ranging from ∼2% in high-income countries to ≥50% in low-income countries, such as Eritrea, Timor Leste, and Burundi (5). In addition to its variability among countries, stunting prevalence also shows important within-country inequalities according to household wealth, maternal education, and place of residence (1, 6). In terms of wealth, data from national surveys carried out since 2000 show that stunting prevalence is ∼2.5 times higher in the poorest quintile of households compared with the richest quintile (1).

Global stunting prevalence decreased from 40% to 23% from 1990 to 2016, representing an annual average reduction of 0.65 percentage points, equivalent to an annual average rate of reduction of 2.1% (5, 7). The national prevalence is highest in South Asian and sub-Saharan African countries (1). The available time series suggests that there has been an acceleration in the speed of stunting reduction globally when comparing the 1990–2000 period against the 2005–2016 time period. What remains unclear is whether different subpopulations have benefited from this increased rate of decline in the same way, and limited information is available on time trends disaggregated by socioeconomic characteristics of children and their families (8, 9). Restrepo-Mendez et al. (8) and Wagstaff et al. (9) analyzed 25 (from 1993 to 2012) and 53 (from 1990 to 2011) countries, respectively. These authors reported mixed results, with most countries failing to show a decline in inequalities; no attempt was made to derive trends on the basis of pooled data from multiple countries.

Data availability has increased markedly in recent years, and in the present analyses we aim to describe global, pooled time trends according to household wealth and place of residence in 67 low- and middle-income countries (LMICs) with ≥2 nationally representative surveys since the mid-1990s. Our analyses respond to the SDGs recommendation for stratifying national results by income and place of residence, in order to document whether poor and rural populations are benefiting from national-level progress in health and nutrition.

## Methods

### 

#### Data source and variables

The present analyses were carried out based on data from Demographic Health Surveys (DHSs) (10) and Multiple Indicators Clusters Surveys (MICSs) (11). These surveys have measured reproductive, maternal, neonatal, and child health and nutrition indicators for >20 y in LMICs. They adhere to the fundamentals of scientific sampling, allowing for representative estimates of the populations sampled, and use standardized data collection procedures, including for anthropometric measurements (12). The International Center for Equity in Health database compiled >300 publicly available MICS and DHS data sets in order to produce disaggregated analyses. The present analyses are limited to 217 nationally representative surveys from 67 countries where stunting estimates were available for ≥2 time points between 1993 and 2015.

In each survey, anthropometric measures were obtained for under-5 children, and additional information including birth date, sex, and data to generate wealth scores was obtained via questionnaires administered through interviews with their primary caretaker. We also included 20 DHSs carried out between 1993 and 1999, which collected data solely for children <3 y of age (under-3). For these surveys, under-5 prevalence was predicted on the basis of the under-3 prevalence by using linear regression in another 150 DHSs with data on prevalence for both age groups. The prevalence of stunting among infants is considerably lower than for children aged 1–4 y (**Supplemental Table 1**). Because infants make up approximately one-third of under-3 children, but only approximately one-fifth of those under-5, the prevalence in the former is ∼3 percentage points greater than among the latter (**Supplemental Table 2**). This difference is most marked in the poorest quintile, so that in order to avoid biasing the results on time trends, it was necessary to estimate under-5 prevalence on the basis of under-5 prevalence for surveys with information on both age groups (13). The best-fit equation was as follows: under-5 (%) = −0.0114274 + 1.104429 × under-3 (%). Prevalence levels in under-3 and under-5 children were highly correlated (Pearson's correlation coefficient = 0.9), and 97% of the variability in under-5 prevalence was explained by under-3 prevalence (adjusted *R*^2^ = 0.97) (**Supplemental Figure 1**).

In both the MICSs and DHSs, standard length and height measuring boards were used to measure recumbent length for children <2 y old and standing height for the remaining under-5 children. Stunting prevalence was calculated as the proportion of children measured whose height or length for age was >2 SDs below the median age- and sex-specific value of the WHO Child Growth Standards (14).

Wealth quintiles and place of residence were the stratification variables. Wealth quintiles are based on scores derived by using principal components analyses, which are applied to a list of household assets and characteristics of the houses. The first quintile (quintile 1) represents the poorest 20% of the population, and the last quintile (quintile 5) represents the wealthiest 20% of the population. Quintiles correspond to the relative position of households within each national sample, rather than absolute income for which data are not available for most studies. The list of assets is specific to each survey and may change over time in the same country because newly introduced assets may become more common. Because fertility is higher among the poor, the poorest quintile tends to include >20% of all children surveyed, whereas the richest quintiles include less than one-fifth of all children. Urban and rural residence was classified according to boundaries provided by local authorities.

#### Data analyses

Stunting prevalence trends were calculated at the group level by using linear multilevel regression models in which surveys were the first-level units and countries (because we had multiple surveys per country) the second-level units. Trends were calculated for all children and for subgroups defined by wealth quintiles and by area of residence (urban or rural) in each survey. Two sets of comparisons were made: *1*) the poorest 20% compared with the remaining 80% and *2*) the poorest 40% compared with the remaining 60%. Our analyses were weighted by the number of under-5 children in each country in 2006 (the median year for all data sets). All of the models were tested for departure from linearity through comparisons between the models including *1*) only the linear term for year and *2*) the linear plus quadratic term.

Analyses were initially carried out for all countries with data, and later stratified by World Bank income groups as of 2015 (low-income and middle-income countries). Ratios of the estimated slopes among poorest and richest groups, as well as rural and urban populations, were calculated to assess differentials.

Additional analyses of wealth inequalities assessed average annual changes in the Slope Index of Inequality (SII) and the Concentration Index of Inequality (CIX). Unlike ratios of rich to poor, both indexes take into account the prevalence of stunting in the 5 quintiles, rather than being limited to a comparison between the poorest and wealthiest groups. They express, respectively, absolute and relative socioeconomic inequalities in stunting, and it is recommended that both indexes are described together in inequality analysis. The SII relies on logistic regression to express in percentage points the absolute difference in stunting prevalence between the extremes of the spectrum of wealth. The CIX expresses on a scale from −1 to +1 how far the observed distribution of stunting is from perfect equality (i.e., when prevalence is the same in all 5 quintiles). In this case, the CIX would be equal to zero. For stunting prevalence, both measures tend to be negative, indicating higher prevalence among the poor. Further information on how to calculate and interpret these indexes is available elsewhere (15). The CIX and SII are calculated from data on individual children; for the 20 surveys limited to children under-3, the summary indexes were based on this age group. In a validation exercise with the use of data from the 150 surveys with information on under-3 and under-5 children, we found almost perfect Pearson correlations (0.97) both for CIX and SII calculated for the 2 age ranges (**Supplemental Figure 2**).

#### Ethical aspects

All of the current analyses were based on data that are publicly available. Ethical clearance was the responsibility of the institutions that administered the surveys.

## Results

Data on time trends stratified by wealth and place of residence were available from 217 surveys in 67 countries, distributed into 96 surveys in 27 low-income countries, 72 surveys in 24 lower middle–income countries, and 49 surveys in 16 upper middle–income countries. These represented 87% of low-income, 46% of lower middle–income, and 29% of upper middle–income countries. Given the small number of countries in the upper middle–income group, we pooled these with lower middle–income countries. **Supplemental Table 3** shows the stunting prevalence in the surveys available for analyses. These ranged from 61.4% in Nepal (1996) to 4.9% in Macedonia (2011).

Stunting prevalence is declining globally and is stratified by country income groupings. On the basis of multilevel linear models, in all 67 countries, the predicted mean prevalence was 50.0% in 1993 and 34.4% in 2014, corresponding to an annual reduction of 0.74 percentage points. The mean prevalence in 1993 was 53.7% in low- and 48.2% in middle-income countries, with annual average linear declines of 0.76 and 0.72 percentage points, respectively.

Analyses of linear trends by wealth quintile showed that the 2 poorest quintiles had similar slopes and prevalence levels in most countries, so that we compared these 2 quintiles (quintiles 1 and 2, or the poorest 40% of households) with the remaining 3 quintiles (quintiles 3–5). Similar slopes of decline were found for the poorest 40% and the remaining 60% within both low-income and middle-income countries (**Table 1**). Moreover, a similar pattern emerged from the comparison of the poorest 20% with the richest 80% (**Supplemental Table 4**).

**TABLE 1:** Time trends in stunting prevalence comparing the 2 poorest (Q1–Q2) with the 3 wealthiest (Q3–Q5) quintiles by country income groups: 1993–2014^1^

		Average slope			
				Ratio of	
Income group	Average			Q1–Q2:	
(World Bank)	national slope	Q1–Q2	Q3–Q5	Q3–Q5	*P* ^2^
Global	−0.74 ± 0.08	−0.78 ± 0.09	−0.74 ± 0.10	1.05	0.758
Low-income	−0.76 ± 0.13	−0.72 ± 0.11	−0.79 ± 0.14	0.91	0.539
Middle-income	−0.72 ± 0.10	−0.82 ± 0.14	−0.68 ± 0.14	1.21	0.860
*P* ^3^	0.725	0.592	0.539		

^1^Values are slopes ± SEs unless otherwise indicated. Slopes are based on linear regressions of stunting prevalence over year of the survey and expressed in percentage points. Q, quintile.

^2^ *P* values for interactions between wealth groups (Q1–Q2, Q3–Q5) and year of the surveys (expressed as slopes).

^3^ *P* values for interactions between country income groupings (low, middle) and year of the survey (expressed as slopes).

**FIGURE 1:**
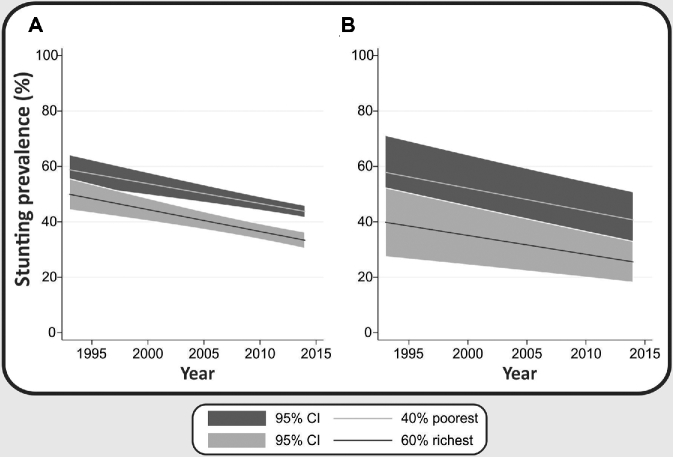
Annual changes in stunting prevalence in the poorest 40% and richest 60% in low-income (A) and middle-income (B) countries.

Figure 1, based on the regression models presented in Table 1, shows the decline in stunting prevalence in both country income groupings, as well as the gap between poorest and richest wealth quintiles. The gap between the poorest 40% and the richest 60% seems to remain stable in both country income groupings.

There was evidence of a significant acceleration in stunting decline for the richest 60%, when all countries were analyzed together. In all other subgroup analyses, quadratic terms indicating acceleration were not significant (**Supplemental Table 5**).

Values of the SII and CIX, which take the whole wealth distribution into account, were negative in 213 of 217 surveys, indicating that poor children were more likely to be stunted. The exceptions were surveys from Armenia (2005), Bosnia and Herzegovina (2011), Kyrgyzstan (2005), and Montenegro (2013). For all countries combined, the SII and the CIX remained stable over time (−30.0% in 1993 and −28.6% in 2014 and 13.2% in 1993 and −13.9% in 2014, respectively). Analyses stratified by country income groupings showed that, although both SII and CIX remained stable in middle-income countries, both relative and absolute inequalities increased over time in low-income countries (**Table 2**), where the SII changed from −19.3% in 1993 to −23.7% in 2014 and the CIX from −6.2% to −10.8% in the same period (**Supplemental Figure 3**).

**TABLE 2:** Time trends in the SII and CIX by country income groups: 1993–2014^1^

	SII		CIX	
Income group (World Bank)	Slope ± SE	*P* ^2^	Slope ± SE	*P* ^2^
Global	0.06 ± 0.17	0.690	0.04 ± 0.16	0.822
Low-income	−0.21 ± 0.09	<0.001	−0.22 ± 0.05	<0.001
Middle-income	0.26 ± 0.29	0.371	−0.07 ± 0.28	0.807
*P* ^3^	0.135		0.322	

^1^Slopes are based on linear regressions of SII and CIX over year of the survey and expressed in percentage points. CIX, Concentration Index of Inequality; SII, Slope Index of Inequality.

^2^ *P* values for slope being different from zero.

^3^ *P* values for interactions between country income groupings (low, middle) and year of the survey (expressed as slopes).

**FIGURE 2:**
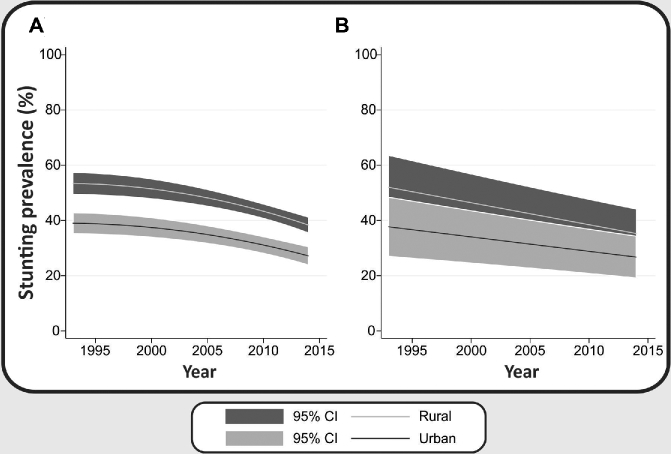
Annual changes in stunting prevalence according to rural and urban areas in low-income (A) and middle-income (B) countries.

When all countries were pooled, declines in stunting prevalences were larger in rural areas than in urban areas (*P* = 0.023), suggesting that the urban-rural gap is narrowing (**Table 3**). This was also observed when LMICs were analyzed separately (Table 3, **Figure 2**), but urban and rural differences were not significant. Tests for departure from linearity were significant for both rural and urban children in low-income countries (Figure 2, **Supplemental Table 6**). An acceleration was also observed for urban children when all countries were combined, consistent with what was found for the richest 60%, as described above.

**TABLE 3:** Trends in urban and rural areas in stunting prevalence by country income groups: 1993–2014^1^

		Average slope			
Income group	Average			Ratio of rural:	
(World Bank)	national slope	Rural	Urban	urban slopes	*P* ^2^
Global	−0.74 ± 0.08	−0.78 ± 0.09	−0.55 ± 0.08	1.42	0.023
Low-income	−0.76 ± 0.13	−0.75 ± 0.13	−0.57 ± 0.11	1.31	0.181
Middle-income	−0.72 ± 0.10	−0.80 ± 0.13	−0.52 ± 0.11	1.52	0.081
*P* ^3^	0.725	0.829	0.712		

^1^Values are slopes ± SEs unless otherwise indicated. Slopes are based on linear regressions of stunting prevalence over year of the survey and expressed in percentage points. Q, quintile.

^2^ *P* values for interactions between wealth groups (rural, urban) and year of the surveys (expressed as slopes).

^3^ *P* values for interactions between country income groupings (low, middle) and year of the survey (expressed as slopes).

## Discussion

Our analyses confirm the results of previous time series (1, 5, 7, 8) that show that global stunting prevalence is decreasing over time. Our estimate of an average annual reduction of 0.74 percentage points is in line with previous results.

Our analyses go beyond earlier publications, which assessed trends on stunting inequalities (8, 9), by pooling results across countries by using multilevel modeling, rather than examining one country at a time. Moreover, we are not aware of previous analyses in which national trends were stratified by country income groups. With 217 surveys from 67 countries, we could investigate national-level progress and inequalities separately in low-income and middle-income countries. We also compared trends in the rural-urban and poor-rich gaps, as well as summary indexes of wealth inequality.

We observed progress in the reduction in the stunting prevalence in the 2 groups of countries. There was evidence of an acceleration in the stunting prevalence in recent years for both rural and urban children from low-income countries. In all other stratified analyses, there was no significant evidence of acceleration.

Taken at face value, annual declines appeared to be slower among the poorest 40% than for the richest 60% in low-income countries, whereas the opposite was observed in middle-income countries (Table 1). However, the differences in slopes were not significant. Statistical power is increased by using summary indexes of inequality, which consider trends in the 5 quintiles instead of only 2 socioeconomic groups (Table 2); these analyses provided significant evidence of an increase in both absolute and relative stunting inequality in low-, but not in middle-income countries.

In a previous analysis of within-country inequalities, Restrepo-Mendez et al. (8) studied 25 countries and found that significant reductions in absolute (SII) and relative (CIX) inequality in stunting prevalence were observed in only 7 and 4 countries, respectively. Wagstaff et al. (9) carried out separate analyses for 53 countries, showing that the median decline in stunting was slightly faster among the richest 60% than in the poorest 40%. They concluded that, overall, absolute and relative inequalities were constant in LMICs. By expanding the number of countries studied and presenting separate results for low-income and middle-income countries, we were able to document significant increases in inequalities among the former.

We also found evidence that the decline in stunting was faster for rural than for urban children (Table 3). We were unable to locate any previous studies on the evolution of urban–rural inequalities in stunting prevalence. Because there are only 2 groups of places of residence, summary indexes cannot be calculated.

The present analyses have limitations. Twenty DHSs reported on stunting prevalence on the basis of under-3 children, instead of under-5 children. Considering that stunting prevalence in children aged 4 and 5 y tends to be higher than in under-3 children (13), we opted to adjust prevalence on the basis of a regression equation derived from 150 DHSs, for which it was possible to calculate under-3 and under-5 prevalence. A second limitation is the absence of information for several countries. Our results are more representative for low-income (87% of which were included) than for middle-income (37%) countries. Last, some populous countries did not contribute with recent data, such as China (for which no data were available), India (latest survey with available data sets for analyses was in 2005), and Brazil (latest survey in 2006). The inclusion of more recent results from these countries may modify the observed patterns in our population-weighted analyses.

Among the strengths of our analyses, we highlight the large number of countries and surveys compared with previous analyses, the consistent methodology and measurement protocols used in the different surveys, and the presentation of trends in relative and absolute summary indexes of inequality, as well as of time trends by wealth and residence.

Scaling-up child survival interventions to reach universal coverage is estimated to reduce two-thirds of under-5 deaths in the world (1). In contrast, scaling-up nutrition-specific interventions is estimated to prevent only approximately one-third of cases of stunting (15). Absolute levels of wealth are strongly associated with stunting prevalence (16). Strategies for reducing stunting and other forms of malnutrition, as proposed in the SDG 2, will depend on, in addition to delivering specific interventions, acting upon the social determinants of health and nutrition. Regular, equity-sensitive monitoring of nutritional status is an essential tool to assess progress and to identify groups that are being left behind. Examples of countries such as Peru (17) and Brazil (18), which managed to reduce stunting prevalence and close the equity gap, show that concerted actions against undernutrition as part of an antipoverty agenda are effective in relatively short periods of time.

## Supplementary Material

Supplemental data

## Notes

Supported by funds from UNICEF, the Wellcome Trust (grant 101815/Z/13/Z), the Bill & Melinda Gates Foundation (grant OPP1135522), and Associação Brasileira de Saúde Coletiva.

Supplemental Tables 1–6 and Supplemental Figures 1–3 are available from the “Supplementary data” link in the online posting of the article and from the same link in the online table of contents at https://academic.oup.com/jn/.

**Abbreviations used:**
CIX: Concentration Index of Inequality
DHS: Demographic Health Surveys
LMIC: low- and middle-income country
MICS: Multiple Indicators Clusters Survey
SDG: Sustainable Development Goal
SII: Slope Index of Inequality
under-3: under the age of 3 y
under-5: under the age of 5 y.
