# Supplementary material for: Socioeconomic Inequalities Persist Despite Declining Stunting Prevalence in Low- and Middle-Income Countries
Source: J Nutr. 2018 Feb 27;148(2):254–8. doi: 10.1093/jn/nxx050 (PMC6084584; doi:10.1093/jn/nxx050)
Supplement: Supplemental data [file nxx050_supp.docx]

**Supplemental Table 1. Stunting prevalence by age in months, in 150 Demographic and Health surveys with information on children under five years.**

| Wealth quintiles | Stunting prevalence (%) | | | | |
| --- | --- | --- | --- | --- | --- |
|  | 0-11m | 12-23m | 24-35m | 36-47m | 48-59m |
| Q1 (poorest) | 22.4 | 45.1 | 52.2 | 50.3 | 45.6 |
| Q2 | 19.5 | 40.4 | 46.1 | 44.4 | 40.5 |
| Q3 | 17.2 | 35.2 | 41.6 | 39.4 | 35.1 |
| Q4 | 11.8 | 30.9 | 35.0 | 32.0 | 28.1 |
| Q5 (wealthiest) | 15.4 | 22.7 | 24.1 | 21.9 | 17.8 |
| All children | 17.8 | 36.0 | 41.4 | 39.1 | 34.8 |

**Supplemental Table 2. Stunting prevalence in children under-three and under-five years by wealth quintile, in 150 surveys with information on the two age ranges.**

| Wealth quintiles | Stunting prevalence (%) | |
| --- | --- | --- |
|  | <3y | <5y |
| Q1 (poorest) | 39.6 | 42.9 |
| Q2 | 35.1 | 38.0 |
| Q3 | 31.1 | 33.5 |
| Q4 | 27.0 | 28.2 |
| Q5 (wealthiest) | 20.0 | 19.7 |
| All children | 31.5 | 33.6 |

#

# **Supplemental Figure 1. Comparison between stunting prevalence in children under three and under five years of age in 150 Demographic and Health Surveys (Pearson’s correlation coefficient = 0.9; adjusted *R^2^*=0.97) (A), also showing Bland and Altman chart (B) ^(1)^.**

**
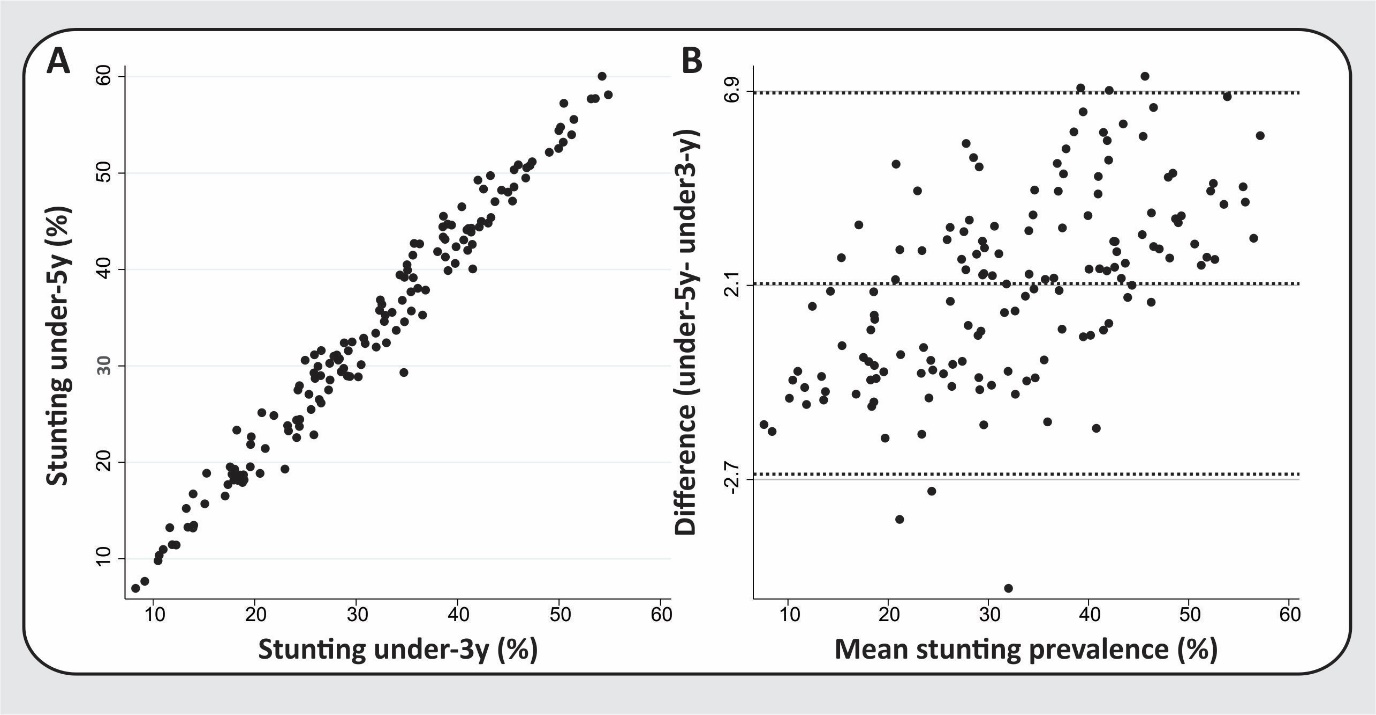
**

# **Supplemental Figure 2. Comparisons between slope index of inequality (SII) (A) and concentration index (CIX) (B) for under-three and under-five stunting prevalence in 150 Demographic and Health Surveys (Pearson’s correlation coefficient = 0.97).**

**
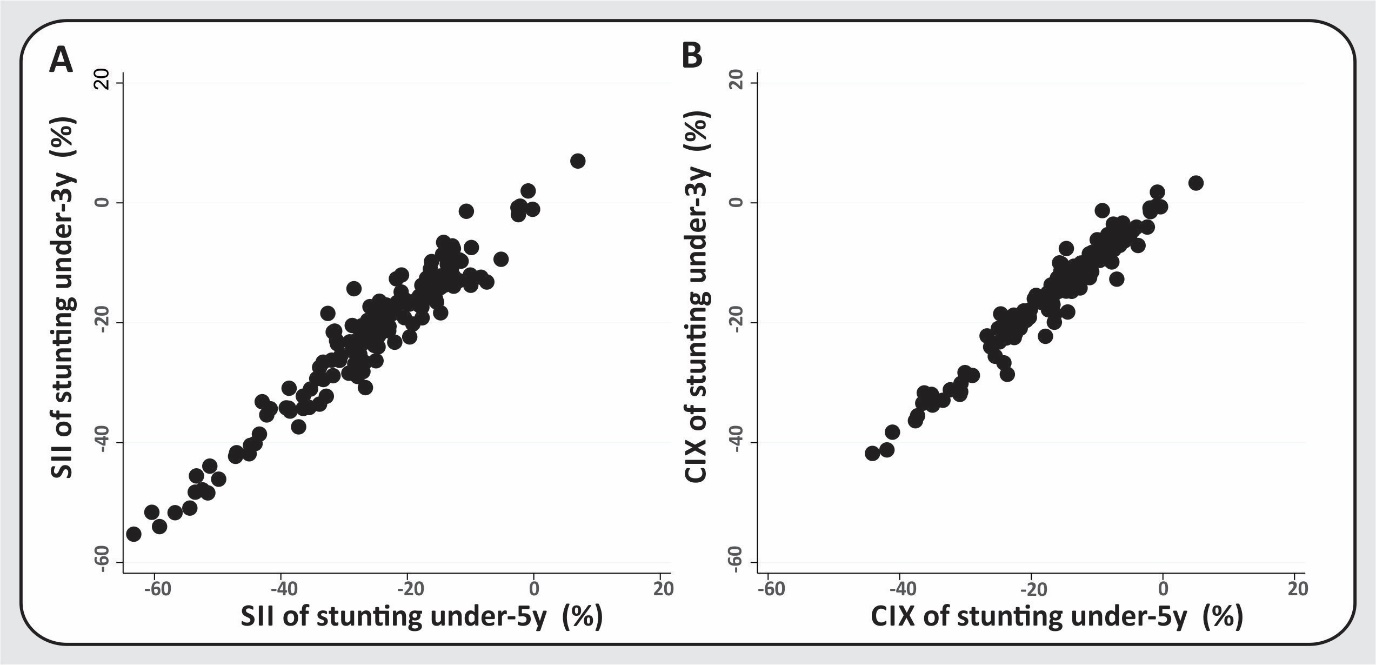
**

# **Supplemental Table 3. List of countries and surveys year included in the analyses according to World Bank income groups, showing stunting prevalence (%).**

| **Country** | **1^st^**  **survey** | **2^nd^**  **survey** | **3^rd^**  **survey** | **4^th^**  **survey** | **5^th^ survey** | **6^th^**  **Survey** | **7^th^ survey** |
| --- | --- | --- | --- | --- | --- | --- | --- |
| **Low income countries** | | | | | | | |
| Bangladesh | 1996 | 1999 | 2004 | 2007 | 2011 | 2012 |  |
|  | 60.0 | 51.2 | 50.6 | 43.1 | 41.3 | 42.0 |  |
| Benin | 1996* | 2001 | 2006 |  |  |  |  |
|  | 34.7 | 35.8 | 43.1 |  |  |  |  |
| Burkina Faso | 1998 | 2003 | 2006^X^ | 2010 |  |  |  |
|  | 41.5 | 43.4 | 41.9 | 34.6 |  |  |  |
| CAR | 1994* | 2006^X^ | 2010^X^ |  |  |  |  |
|  | 42.1 | 44.2 | 40.7 |  |  |  |  |
| Cambodia | 2000 | 2005 | 2010 | 2014 |  |  |  |
|  | 49.7 | 42.7 | 39.9 | 32.4 |  |  |  |
| Chad | 1996 | 2004 | 2010^X^ |  |  |  |  |
|  | 44.6 | 44.7 | 38.7 |  |  |  |  |
| Comoros | 1996* | 2012 |  |  |  |  |  |
|  | 41.0 | 30.1 |  |  |  |  |  |
| Congo Democratic Republic | 2007 | 2010^X^ | 2013 |  |  |  |  |
|  | 45.5 | 43.4 | 42.7 |  |  |  |  |
| Ethiopia | 2000 | 2005 | 2011 |  |  |  |  |
|  | 57.7 | 50.9 | 44.4 |  |  |  |  |
|  |  |  |  |  |  |  |  |
| Gambia | 2005^X^ | 2013 |  |  |  |  |  |
|  | 27.7 | 24.5 |  |  |  |  |  |
| Guinea | 1999 | 2005 | 2012 |  |  |  |  |
|  | 30.6 | 39.2 | 31.2 |  |  |  |  |
| Haiti | 1994 | 2000 | 2005 | 2012 |  |  |  |
|  | 36.8 | 28.7 | 29.4 | 21.9 |  |  |  |
| Kenya | 1993 | 1998* | 2003 | 2008 |  |  |  |
|  | 39.9 | 39.2 | 35.7 | 35.3 |  |  |  |
| Liberia | 2007 | 2013 |  |  |  |  |  |
|  | 39.4 | 31.6 |  |  |  |  |  |
| Madagascar | 1997* | 2003 | 2008 |  |  |  |  |
|  | 59.6 | 53.2 | 50.3 |  |  |  |  |
| Malawi | 2000 | 2004 | 2006^X^ | 2010 | 2013^X^ |  |  |
|  | 54.4 | 52.2 | 53.1 | 47.1 | 42.4 |  |  |
| Mali | 1995* | 2001 | 2006 |  |  |  |  |
|  | 38.7 | 41.9 | 37.7 |  |  |  |  |
| Mozambique | 1997* | 2003 | 2008^X^ | 2011 |  |  |  |
|  | 45.6 | 47.0 | 43.7 | 42.6 |  |  |  |
| Nepal | 1996* | 2001 | 2006 | 2011 | 2014^X^ |  |  |
|  | 61.4 | 57.2 | 49.3 | 40.5 |  |  |  |
| Niger | 1998* | 2006 | 2012 |  |  |  |  |
|  | 50.2 | 54.8 | 43.9 |  |  |  |  |
|  |  |  |  |  |  |  |  |
| Rwanda | 2000 | 2005 | 2010 |  |  |  |  |
|  | 48.3 | 50.8 | 44.3 |  |  |  |  |
| Sierra Leone | 2005^X^ | 2008 | 2010^X^ | 2013 |  |  |  |
|  | 47.0 | 36.4 | 44.4 | 37.9 |  |  |  |
| Tajikistan | 2005 | 2012 |  |  |  |  |  |
|  | 33.1 | 26.2 |  |  |  |  |  |
| Tanzania | 1996 | 1999 | 2004 | 2010 |  |  |  |
|  | 49.5 | 48.2 | 44.3 | 42.0 |  |  |  |
| Togo | 1998* | 2006^X^ | 2010^X^ | 2013 |  |  |  |
|  | 30.6 | 31.1 | 29.7 | 27.5 |  |  |  |
| Uganda | 1995 | 2000 | 2006 | 2011 |  |  |  |
|  | 45.0 | 44.4 | 38.1 | 33.4 |  |  |  |
| Zimbabwe | 1994* | 1999 | 2005 | 2009^X^ | 2010 | 2014^X^ |  |
|  | 30.5 | 32.4 | 34.6 | 35.1 | 32.0 | 27.6 |  |
| **Lower middle income countries** | | | | | | | |
| Armenia | 2000 | 2005 | 2010 |  |  |  |  |
|  | 16.7 | 17.9 | 19.3 |  |  |  |  |
| Bolivia | 1994* | 1998 | 2003 | 2008 |  |  |  |
|  | 36.6 | 32.9 | 32.3 | 27.1 |  |  |  |
| Cameroon | 1998* | 2004 | 2006^X^ | 2011 |  |  |  |
|  | 38.9 | 35.6 | 36.3 | 32.5 |  |  |  |
| Congo Brazzaville | 2005 | 2011 |  |  |  |  |  |
|  | 30.8 | 24.4 |  |  |  |  |  |
| Cote dIvoire | 1994* | 1998 | 2006^X^ | 2011 |  |  |  |
|  | 32.9 | 31.0 | 40.1 | 29.7 |  |  |  |
| Egypt | 1995 | 2005 | 2008 | 2014 |  |  |  |
|  | 33.7 | 22.9 | 28.9 | 21.4 |  |  |  |
| Ghana | 1993* | 1998 | 2003 | 2006^X^ | 2008 | 2011 | 2014 |
|  | 34.7 | 30.6 | 35.3 | 28.4 | 28.0 | 22.7 | 18.8 |
| Guatemala | 1995 | 1998 | 2008 |  |  |  |  |
|  | 55.6 | 54.0 | 48.0 |  |  |  |  |
| Guyana | 2006^X^ | 2009 |  |  |  |  |  |
|  | 18.0 | 18.2 |  |  |  |  |  |
| Honduras | 2005 | 2011 |  |  |  |  |  |
|  | 30.0 | 22.7 |  |  |  |  |  |
| India | 1998* | 2005 |  |  |  |  |  |
|  | 55.0 | 48.0 |  |  |  |  |  |
| Kyrgyzstan | 1997* | 2005^X^ | 2012 | 2014^X^ |  |  |  |
|  | 34.6 | 18.1 | 17.7 | 12.9 |  |  |  |
| Lao | 2006^X^ | 2011^X^ |  |  |  |  |  |
|  | 47.6 | 44.2 |  |  |  |  |  |
| Lesotho | 2004 | 2009 |  |  |  |  |  |
|  | 44.1 | 39.2 |  |  |  |  |  |
| Mauritania | 2007^X^ | 2011^X^ |  |  |  |  |  |
|  | 31.4 | 29.7 |  |  |  |  |  |
|  |  |  |  |  |  |  |  |
| Moldova | 2005 | 2012^X^ |  |  |  |  |  |
|  | 10.4 | 6.4 |  |  |  |  |  |
| Mongolia | 2005^X^ | 2010^X^ |  |  |  |  |  |
|  | 27.5 | 15.3 |  |  |  |  |  |
| Nicaragua | 1998 | 2001 |  |  |  |  |  |
|  | 30.3 | 24.9 |  |  |  |  |  |
| Nigeria | 2003 | 2007^X^ | 2008 | 2011^X^ | 2013 |  |  |
|  | 42.4 | 42.8 | 40.6 | 35.8 | 36.8 |  |  |
| Senegal | 2005 | 2010 | 2012 | 2014 |  |  |  |
|  | 19.3 | 26.5 | 18.7 | 18.7 |  |  |  |
| State of Palestine | 2010^X^ | 2014^X^ |  |  |  |  |  |
|  | 10.9 | 7.4 |  |  |  |  |  |
| Swaziland | 2006 | 2010^X^ |  |  |  |  |  |
|  | 28.9 | 30.9 |  |  |  |  |  |
| Uzbekistan | 1996* | 2006^X^ |  |  |  |  |  |
|  | 37.5 | 19.6 |  |  |  |  |  |
| Zambia | 1996 | 2001 | 2007 | 2013 |  |  |  |
|  | 48.6 | 52.6 | 45.4 | 40.1 |  |  |  |
| **Upper middle income countries** | | | | | | | |
| Albania | 2005^X^ | 2008 |  |  |  |  |  |
|  | 26.7 | 19.3 |  |  |  |  |  |
| Belize | 2006^X^ | 2011^X^ |  |  |  |  |  |
|  | 22.5 | 19.3 |  |  |  |  |  |
| Bosnia and Herzegovina | 2006^X^ | 2011^X^ |  |  |  |  |  |
|  | 11.8 | 8.9 |  |  |  |  |  |
| Brazil | 1996 | 2006 |  |  |  |  |  |
|  | 13.3 | 6.2 |  |  |  |  |  |
| Colombia | 1995 | 2000 | 2005 | 2010 |  |  |  |
|  | 19.5 | 18.2 | 15.7 | 13.2 |  |  |  |
| Dominican Republic | 1996 | 2002 | 2007 | 2013 |  |  |  |
|  | 13.5 | 11.4 | 9.8 | 6.9 |  |  |  |
| Gabon | 2000 | 2012 |  |  |  |  |  |
|  | 25.5 | 16.5 |  |  |  |  |  |
| Jordan | 1997 | 2002 | 2012 |  |  |  |  |
|  | 11.0 | 11.5 | 7.7 |  |  |  |  |
| Kazakhstan | 1995* | 1999 | 2006^X^ | 2010^X^ |  |  |  |
|  | 21.4 | 13.2 | 17.5 | 13.1 |  |  |  |
| Macedonia | 2005^X^ | 2011^X^ |  |  |  |  |  |
|  | 11.5 | 4.9 |  |  |  |  |  |
| Montenegro | 2005^X^ | 2013^X^ |  |  |  |  |  |
|  | 8.0 | 9.4 |  |  |  |  |  |
| Namibia | 2000 | 2006 | 2013 |  |  |  |  |
|  | 29.0 | 29.0 | 23.7 |  |  |  |  |
| Peru | 1996 | 2000 | 2005/2012^#^ |  |  |  |  |
|  | 31.6 | 31.1 | 29.3/18.1 |  |  |  |  |
|  |  |  |  |  |  |  |  |
| Serbia | 2005^X^ | 2010^X^ | 2014^X^ |  |  |  |  |
|  | 8.2 | 6.6 | 6.0 |  |  |  |  |
| Suriname | 2006^X^ | 2010^X^ |  |  |  |  |  |
|  | 10.7 | 8.8 |  |  |  |  |  |
| Turkey | 1993^X^ | 1998^X^ | 2003^X^ |  |  |  |  |
|  | 23.3 | 18.9 | 15.2 |  |  |  |  |

* Under-five stunting prevalence based on under-three estimates

^X^ MICS surveys; all other surveys are DHS

^#^ Continuous DHS based on yearly data collection

# **Supplemental Table 4. Time trends in stunting prevalence comparing the poorest (Q1) with the four wealthiest quintiles (Q2-Q5), by country income groups,1993-2014.**

| Income group (World Bank) | Average national slope | Average slope in | | Ratio  Q1/Q2-Q5  Slopes | P* |
| --- | --- | --- | --- | --- | --- |
|  |  | **Q1** | **Q2-Q5** |  |  |
|  | **Slope (SE)** | **Slope (SE)** | **Slope (SE)** |  |  |
| Global | -0.74 (0.08) | -0.73 (0.10) | -0.75 (0.10) | 0.98 | 0.815 |
| Low income | -0.76 (0.13) | -0.62 (0.08) | -0.80 (0.15) | 0.78 | 0.357 |
| Middle income | -0.72 (0.10) | -0.82 (0.18) | -0.70 (0.13) | 1.17 | 0.760 |
| P^#^ | 0.725 | 0.295 | 0.586 |  | |

* P levels for interactions between wealth groups (Q1, Q2-Q5) and year of the surveys (expressed as slopes).

^#^ P level for interaction between country income groupings (low, middle) and year of the survey (expressed as slopes).

Note: Slopes are based on linear regressions of stunting prevalence over year of the survey, and expressed in percent points.

# **Supplemental Table 5. Equations fitted to the data based on multilevel regressions at national level and among the two poorest (Q1-Q2) and the three wealthiest quintiles (Q3-Q5), stratified by country income groups, 1993-2014.**

|  |  | **National** | **Q1-Q2** | **Q3-Q5** |
| --- | --- | --- | --- | --- |
| **All countries** | Intercept | 0.5017388 | 0.5871114 | 0.4104134 |
|  | Year | -0.0074053 (p<0.001) | -0.0077431 (p<0.001) | -0.001752 (p=0.593) |
|  | Year squared | - | - | -0.0002411 (p=0.035) |
| **Low income** | Intercept | 0.5461155 | 0.5950436 | 0.5073005 |
|  | Year | -0.0075768 (p<0.001) | -0.0071549 (p<0.001) | -0.0079059 (p<0.001) |
|  | Year squared | - | - | - |
| **Middle income** | Intercept | 0.4831942 | 0.5867148 | 0.4051476 |
|  | Year | -0.0071455 (p<0.001) | -0.0081774 (p<0.001) | -0.0068129 (p<0.001) |
|  | Year squared | - | - | - |

Note: Quadratic terms were tested in all models, with significant (P<0.05) shown in table.

# **Supplemental Table 6. Equations fitted to the data based on multilevel regressions at national level and among rural and urban population, stratified by country income groups, 1993-2014.**

|  |  | **National** | **Rural** | **Urban** |
| --- | --- | --- | --- | --- |
| **All countries** | Intercept | 0.5017388 | 0.5387937 | 0.3579163 |
|  | Year | -0.0074053 (p<0.001) | - 0.0078011 (p<0.001) | 0.0022205 (p=0.473) |
|  | Year squared | - | - | -0.0003301 (p=0.017) |
| **Low income** | Intercept | 0.5461155 | 0.5371474 | 0.3771135 |
|  | Year | -0.0075768 (p<0.001) | -0.0005207 (p=0.908) | 0.0026115 (p=0.302) |
|  | Year squared | - | -0.0002913 (p=0.047) | -0.0003482 (p=0.001) |
| **Middle income** | Intercept | 0.4831942 | 0.528067 | 0.382179 |
|  | Year | -0.0071455 (p<0.001) | -0.0079686 (p<0.001) | -0.0052213 (p=434) |
|  | Year squared | - | - | - |

Note: Quadratic terms were tested in all models, with significant (P<0.05) shown in table.

# **Supplemental Figure 3. Time trends in the slope index of inequality (SII) and concentration index (CIX) in low-income (A) and middle-income countries (B), 1993-2014.**

**
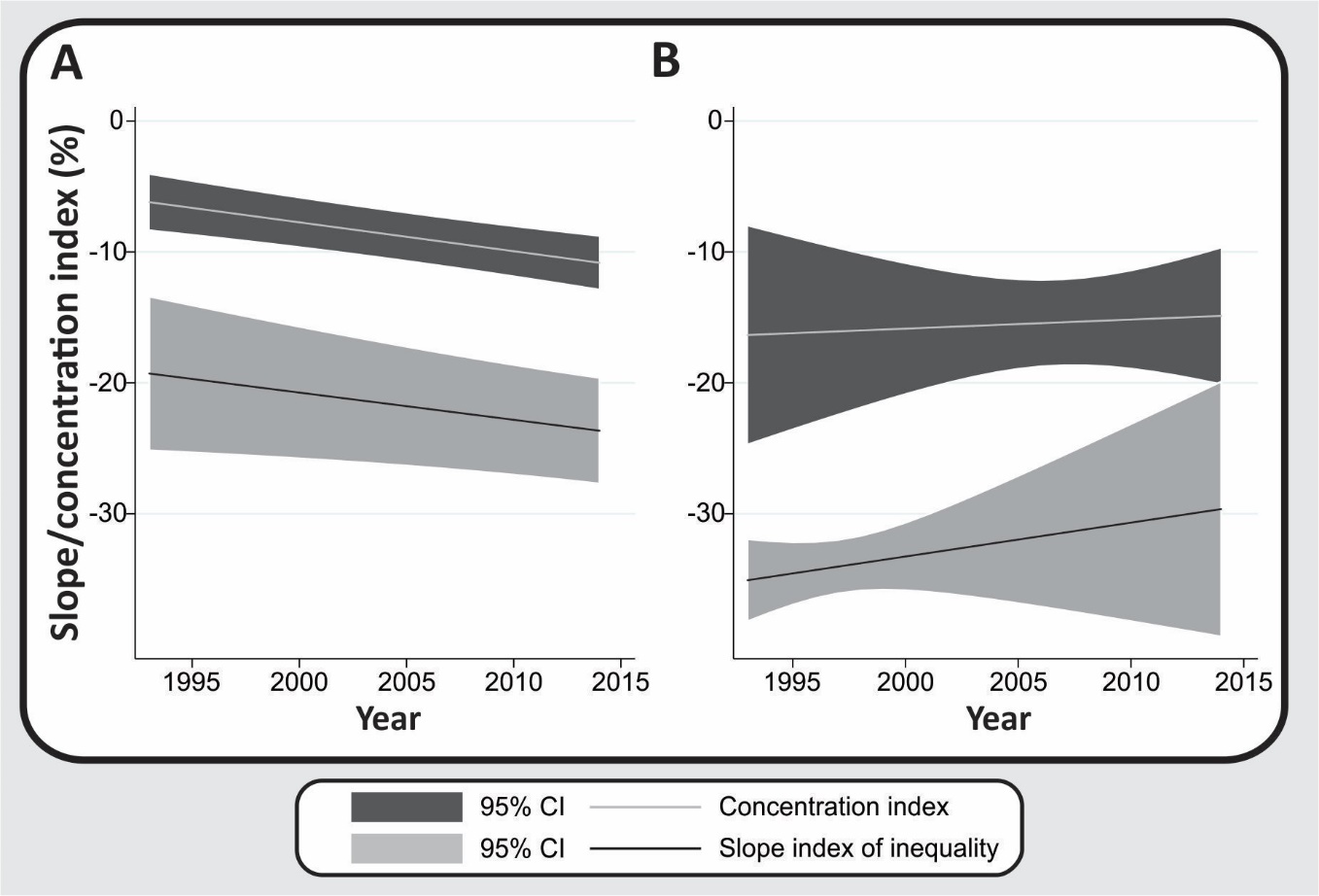
**

Note: Declining lines show increased pro-rich inequalities.

# **Supplemental Reference**

1. Bland JM, Altman DG. Measuring agreement in method comparison studies. Stat Methods Med Res. 1999;8:135–60.
